# Granulocyte Colony-Stimulating Factor Activating HIF-1α Acts Synergistically with Erythropoietin to Promote Tissue Plasticity

**DOI:** 10.1371/journal.pone.0010093

**Published:** 2010-04-09

**Authors:** Shih-Ping Liu, Shin-Da Lee, Hsu-Tung Lee, Demeral David Liu, Hsiao-Jung Wang, Ren-Shyan Liu, Shinn-Zong Lin, Woei-Cherng Shyu

**Affiliations:** 1 Center for Neuropsychiatry, China Medical University Hospital, Taichung, Taiwan; 2 Graduate Institute of Basic Medical Science, China Medical University, Taichung, Taiwan; 3 Department of Physical Therapy, Graduate Institute of Rehabilitation Science, China Medical University, Taichung, Taiwan; 4 Department of Healthcare Administration, Asia University, Taichung, Taiwan; 5 Department of Neurosurgery, Taichung Veterans General Hospital, Taichung, Taiwan; 6 Department of Dentistry, China Medical University Hospital, Taichung, Taiwan; 7 Department of Nuclear Medicine, Taipei Veterans General Hospital, National Yang-Ming University, Taipei, Taiwan; 8 Graduate Institute of Immunology, China Medical University, Taichung, Taiwan; University of Nebraska, United States of America

## Abstract

Stroke and peripheral limb ischemia are serious clinical problems with poor prognosis and limited treatment. The cytokines erythropoietin (EPO) and granulocyte-colony stimulating factor (G-CSF) have been used to induce endogenous cell repair and angiogenesis. Here, we demonstrated that the combination therapy of EPO and G-CSF exerted synergistic effects on cell survival and functional recovery from cerebral and peripheral limbs ischemia. We observed that even under normoxic conditions, G-CSF activates hypoxia-inducible factor-1α (HIF-1α), which then binds to the EPO promoter and enhances EPO expression. Serum EPO level was significantly increased by G-CSF injection, with the exception of Tg-HIF-1α^+f/+f^ mice. The neuroplastic mechanisms exerted by EPO combined with G-CSF included enhanced expression of the antiapoptotic protein of Bcl-2, augmented neurotrophic factors synthesis, and promoted neovascularization. Further, the combination therapy significantly increased homing and differentiation of bone marrow stem cells (BMSCs) and intrinsic neural progenitor cells (INPCs) into the ischemic area. In summary, EPO in combination with G-CSF synergistically enhanced angiogenesis and tissue plasticity in ischemic animal models, leading to greater functional recovery than either agent alone.

## Introduction

Erythropoietin (EPO), a 30.4 kDa glycoprotein, acts mainly on late erythroid precursor cells to induce maturation. Exogenous administration of EPO and endogenous upregulation of EPO receptors have been demonstrated to promote neuronal survival in the injured brain [1]. EPO has also been identified as an effective neuroprotective agent in neuronal cell cultures [2]. Furthermore, granulocyte-colony stimulating factor (G-CSF) is a 19.6 kDa glycoprotein that has long been used to treat neutropenia [3]. Similar to EPO, G-CSF has also shown neuroprotective potential in animal models of stroke [4], [5] and in neuronal cultures [6]. Hypoxia-inducible factor-1α (HIF-1α) is a heterodimeric transcription factor composed of two basic helix-loop-helix (bHLH) proteins of the PAS family, HIF-1α and HIF-1β [7]. Although HIF-1α activation is important in the response to environmental hypoxia by genes encoding vascular endothelial growth factor (VEGF), stromal cell derived factor 1 (SDF-1) and EPO, there is increasing evidence that HIF-1α activation [8] induced by interleukin (IL)-1β and TNF [9], angiotensin II [10], thrombin [11], and insulin [12] also occurs under normoxic conditions. However, to date there have been no reports of G-CSF activating HIF-1α to upregulate EPO expression under normoxic conditions.

Previous reports have described the beneficial effects from exogenous administration of multiple cytokines, such as EPO, G-CSF, stem cell factor (SCF) and IL-11 [13], [14], [15]. The simultaneous administration of two or three cytokines may result in non-additive, synergistic or inhibitory interactions. EPO combined with G-CSF not only rescued patients suffering from life-threatening neutropenia but also improved blood cell regeneration in patients receiving high-dose chemotherapy [16], [17]. However, the neuroplastic and angiogenic effects of combined EPO and G-CSF treatment have not been investigated.

In this study, we intended to demonstrate that G-CSF upregulates EPO expression via the induction of HIF-1α activity, and that the co-treatment of G-CSF and EPO synergistically enhances neural survival and angiogenesis in both primary cortical cultures and animal models of ischemia.

## Results

### G-CSF Stimulated HIF-1α and EPO Expression

**Figure 1:**
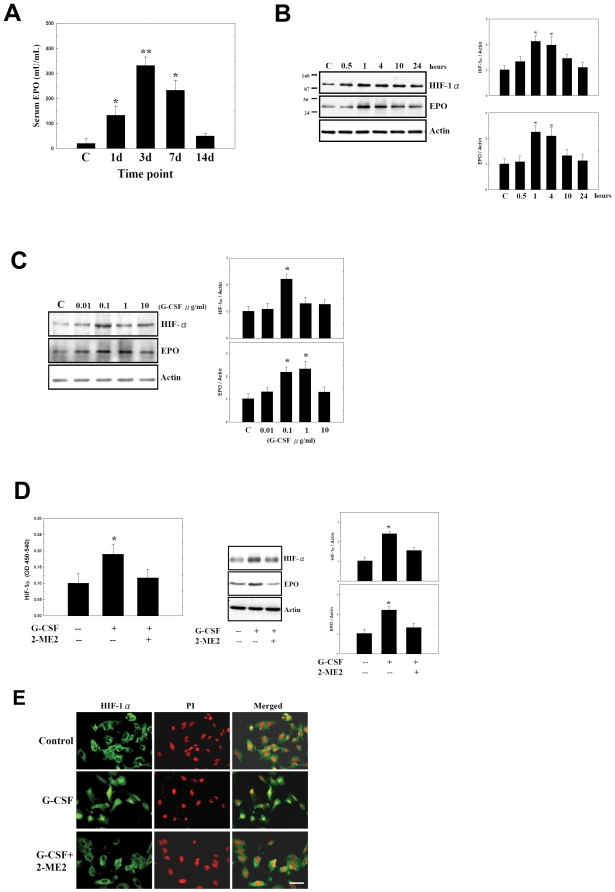
G-CSF increased serum EPO in human and stimulated the expression of EPO by activating HIF-1α in HUVECs. (A) G-CSF-treatment for 5 consecutive days in human showed significant increases serum EPO level compared to control [C]. (B–C) G-CSF induced the protein expression of both HIF-1α and EPO. (D) G-CSF also enhanced the activity of HIF-1α in cell lysate. The upregulation of HIF-1α activity, and protein expression of HIF-1α and EPO stimulated by adding G-CSF returned to normal levels after addition of 2-methoxyestradiol (2-ME2). (E) G-CSF treatment induced the translocation of HIF-1α into nuclei (PI: propidium iodide, nuclear stain) or to perinuclear areas. In contrast, pretreatment with 2-ME2 inhibited the nuclear translocation of HIF-1α. Mean ± SEM, *P<0.05 and **P<0.01 vs. control. Bar  = 50 µm.

To evaluate whether subcutaneous injection of G-CSF for 5 consecutive days could enhance EPO production in humans, serum EPO levels of human donors (*n* = 24) were measured by ELISA at 1, 3, 7, and 14 days. We noted an increase in serum EPO level, which the peak level was seen in the 3th days after first G-CSF injection (Fig. 1A).

Next, to examine increased EPO protein expression from HIF-1α activation, human umbilical vein endothelial cells (HUVECs, Cambrex) were treated with G-CSF for different durations (0.5, 1, 4, 10 and 24 hours), and at different concentrations (0.01, 0.1, 1 and 10 µg/mL). HIF-1α and of EPO protein expression increased following G-CSF treatment (Fig. 1B–C). In addition, G-CSF increased HIF-1α activity in whole cell lysate (Fig. 1D). The protein levels of HIF-1α and EPO stimulated by G-CSF were blocked by pretreatment with 2-methoxyestradiol (2-ME2, 5 µM; Fig. 1D).

The subcellular location of HIF-1α in HUVECs with or without G-CSF treatment was determined by immunohistochemistry. Without G-CSF treatment, HIF-1α was primarily localized in the cytosol (Fig. 1E). G-CSF treatment induced the translocation of HIF-1α into the nucleus or to perinuclear areas (Fig. 1E). Pretreatment with 2-ME2 in HUVECs for 16 hours blocked the HIF-1α nuclear translocation (Fig. 1E).

**Figure 2:**
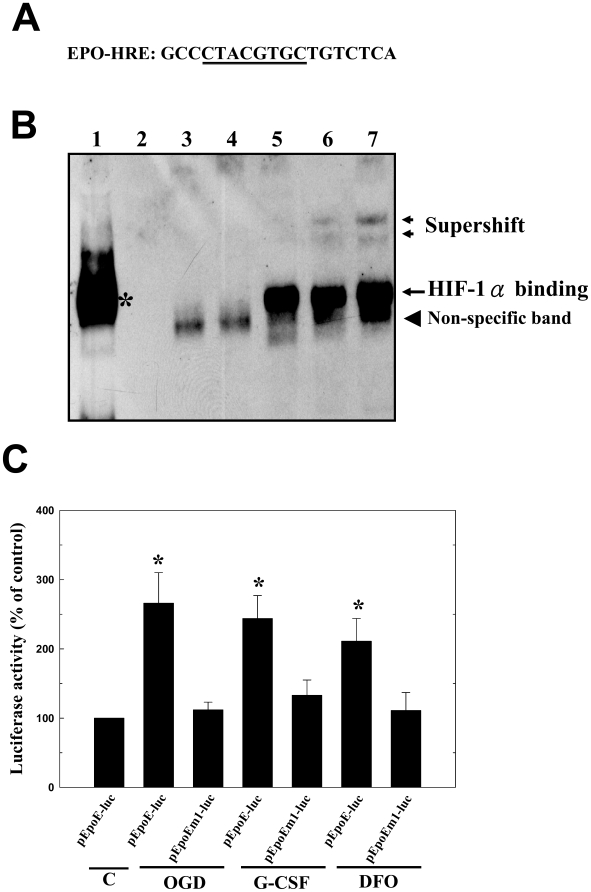
G-CSF promoted HIF-1α transcriptional activity and binding to the HRE of EPO promoter. (A) An oligonucleotide containing the HRE of the HIF-1α-binding sequence (underlined) from the EPO gene (EPO-HRE) was used in EMSA (B), comprising positive control (black star, Lane 1), negative control (Lane 2), and DNA (DIG-labeled oligonucleotide) binding complex (Lane 5; large arrow). Binding was reduced by competition with non-labeled probe (Lane 3, 100X excess; and Lane 4, 300X excess). Nonspecific bands (arrow head) were also shown (including Land 3 and 4). For supershift assay (small arrows), polyclonal anti-HIF-1α (Lane 6) and monoclonal anti-HIF-1α (Lane 7) were used. (C) In a luciferase reporter assay, luciferase activity was higher in the G-CSF-treated cells transfected with pEpoE-luc than that with mutant pEpoEm1-luc, or the control cells. The G-CSF-stimulated luciferase activities were similar to those in oxygen glucose deprivation (OGD) and chemical hypoxia (DFO) conditions. Mean ± SEM, *P<0.05 and **P<0.01 vs. control.

According to results from an electrophoretic mobility shift assay (EMSA) using an 18-bp oligonucleotide probe containing the HRE with the HIF-1α-binding site of the EPO gene promoter (Fig. 2A), G-CSF at a concentration of 0.1 µg/mL was sufficient to induce DNA binding complex formation. Binding was reduced by competition with an unlabeled oligonucleotide, and the complexes were supershifted by a specific HIF-1α antibody in a similar manner in reaction to hypoxia, indicating the presence of this protein in these complexes (Fig. 2B). In addition, oxygen glucose deprivation (OGD), G-CSF (0.1 µg/mL) and chemical hypoxic (deferoxamine, DFO) conditions induced higher luciferase reporter gene activity in a construct (pEpoE-luc) containing HRE from the erythropoietin gene coupled to a SV40 promoter than in a mutant (pEpoEm1-luc) construct or control cells (Fig. 2C).

### EPO+G-CSF Exerted Survival and Plastic Effects

**Figure 3:**
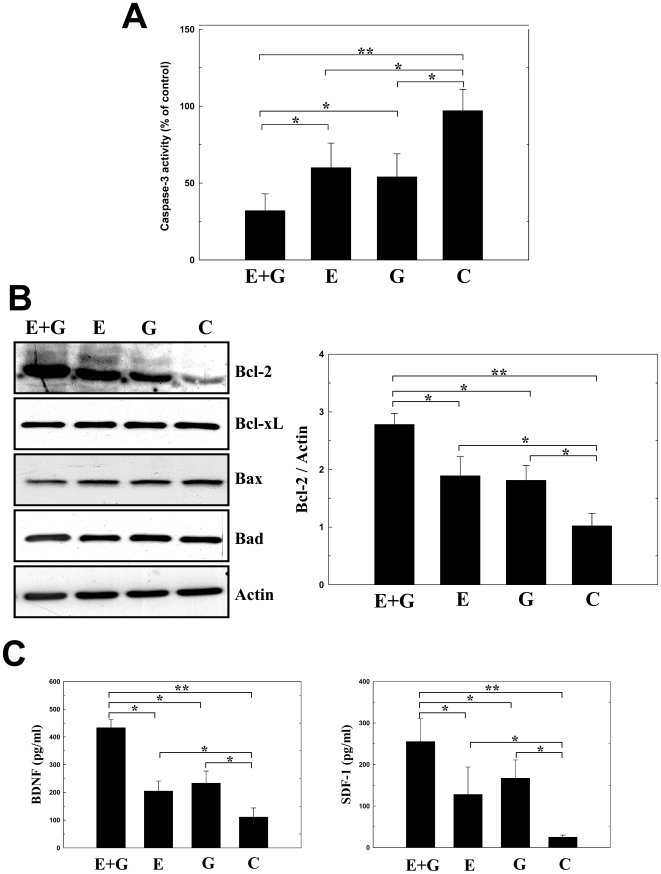
EPO+G-CSF exerted an anti-apoptotic effect and enhanced neurotrophic factor synthesis in primary cortical cultures (PCCs). (A) Under OGD, PCCs pretreated with EPO+G-CSF (E+G) resulted in less caspase-3 activity than with EPO (E) or G-CSF (G) alone, or controls (C). (B) In a Western blot analysis, EPO+G-CSF-treated PCCs expressed more Bcl-2 than EPO, G-CSF or control groups. (C) Measurement of neurotrophic factors by ELISA revealed higher levels of BDNF and SDF-1 in the EPO+G-CSF-treated PCCs than in EPO- or G-CSF-treated cells or the control. Mean ± SEM, *P<0.05 and **P<0.01 vs. control.

To examine whether EPO combined with G-CSF exerts a neuroprotective effect through an antiapoptotic pathway, Bcl-2 protein expression, caspase-3 activity and synthesis of neurotrophic factors under OGD were measured in primary cultures of rat cortical cultures (PCCs) with or without co-treatment of EPO and G-CSF. In OGD, PCCs pretreated with EPO+G-CSF for 12 hours showed significantly less caspase-3 activity by fluorimetric methods than EPO alone, G-CSF alone or control groups (Fig. 3A). Levels of anti-apoptotic Bcl-2 also increased significantly in the EPO+G-CSF group compared with EPO alone, G-CSF alone, and control group (Fig. 3B). PCCs treated with EPO+G-CSF for 3–5 days had higher titers of brain derived neurotrophic factor (BDNF) and stromal cell derived factor 1 (SDF-1) than EPO alone, G-CSF alone, or control groups (Fig. 3C).

**Figure 4:**
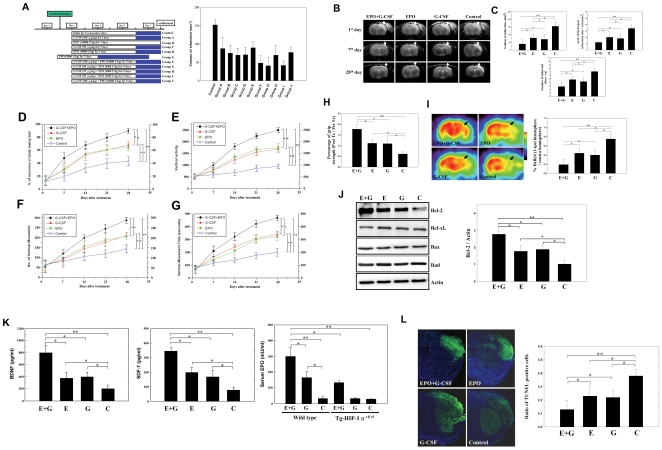
Administration of EPO+G-CSF to cerebral ischemic rats reduced infarct size and improved neurological function. (A) Experimental protocols for determining the best combinations of EPO and G-CSF to reduce infarct size. The dosage, combination and injection duration in each group are indicated in the white rectangles. The blue rectangles in each group represent non-treatment day-point after cytokine injections before euthanasia. In group E, EPO 5000 U/kg treatment started 30 minutes before stroke initiation. Group C (G-CSF alone), group D (EPO alone), group I (EPO+G-CSF) and saline-control group were selected for further study. (B) Representative MRI of ischemic rat brain: the white areas (white arrows) indicate the infarct zone in the right cerebral cortex on the 1^st^, 7^th^ and 28^th^ day after cerebral infarction. (C) Three formats of cerebral infarction assessment including total infarct volume, area of largest infarct section and number of infarct sections at the 7^th^ day after cerebral ischemia were measured in rats treated with EPO+G-CSF (E+G) than in EPO (E) or G-CSF (G)-treated rats or the control (C) group. (D-G) Body asymmetry and locomotor activities after MCA ligation were measured in rats receiving EPO+G-CSF, EPO, G-CSF, and control saline from 7 to 28 days recovery. (H) Forelimb grip strength before and after ischemia were measured in rats receiving EPO+G-CSF, EPO, G-CSF, and control saline. (I) Representative deficit (black arrow, coronal view) and semi-quantitative measurements of [^18^F]fluoro-2-deoxyglucose positron emission tomography (FDG-PET) images of the right cortex of EPO+G-CSF, EPO, G-CSF and control group. (J) Bcl-2, Bcl-xL, Bax, and Bad proteins expression in rats' brain were analyzed 24 hr post-cerebral ischemia following treatment with EPO+G-CSF, EPO, G-CSF or saline. (K) Neurotrophic factors BDNF and SDF-1 level in rats' brain were measured by ELISA following treatment with EPO+G-CSF EPO, G-CSF or saline. Serum EPO levels were measured by ELISA in wild-type mice (C57BL/6 mice) and Tg-HIF-1α^+f/+f^ mice treated with G-CSF and EPO+G-CSF injection. (L) Representative TUNEL (green) and Hoechst 33342 (blue) co-staining images of cells death in right hemisphere of ischemic rat brains from EPO+G-CSF, EPO, G-CSF or control groups. Mean ± SEM, *P<0.05 and **P<0.01 vs. control. Bar  = 50 µm.

According to previous publications, we designed into 11 experimental groups (each group *n = *8) with different therapeutic recipes (Fig. 4A) [1], [6], [16], [18], [19], [20], [21]. At 7 d post-cerebral ischemia, infarct volumes were significantly smaller in three groups: EPO (10,000 U/kg) + G-CSF (100 µg/kg) in Group F; EPO (5000 U/kg) + G-CSF (50 µg/kg) in Group G; and EPO (2500 U/kg) + G-CSF (50 µg/kg) in Group I (Fig. 4A). We selected a lower dosage combination group from one of these three groups to investigate the combination therapeutic effect in comparison with those for single agent and saline control. In all, we selected 4 of the 11 groups (each treatment started at one day after cerebral ischemia) for this study: 1) a low-dose combination of EPO (2500 U/kg) + G-CSF (50 µg/kg) (Group I), 2) G-CSF alone (50 µg/kg) (Group C), 3) EPO alone (5000 U/kg) (Group D), and 4) a saline control (Fig. 4B).

Seven days after cerebral ischemia, infarct volume was significantly reduced from an average of 159±32 mm^3^ in saline-treated controls to 77±26 mm^3^ in EPO, 71±26 mm^3^ in G-CSF, and 42±23 mm^3^ in EPO+G-CSF group (Fig. 4C). The infarcted area of the largest infarcted slice decreased significantly from 17.1±5.3 mm^2^ in controls, to 10.1±4.1 mm^2^ in EPO, 9.0±5.2 mm^2^ in G-CSF, and 6.1±3.9 mm^2^ in EPO+G-CSF group (Fig. 4C). The number of infarcted slices also decreased significantly from 7.1±1.8 slices/rat in control animals to 4.8±1.9 slices/rat in EPO-, 4.3±1.4 slices/rat in G-CSF-, and 2.7±1.3 slices/rat in EPO+G-CSF-treated animals (Fig. 4C).

Body asymmetry, locomotor activity tests and grip strength measurement were used to assess the neurological functional recovery in EPO+G-CSF-treated, EPO-treated, G-CSF-treated and control rats (each group *n = *10). EPO+G-CSF-treated rats showed much higher percentage of recovery in body swing tests than rats treated with EPO only, G-CSF only, or control rats (Fig. 4D). Locomotor activities after cerebral ischemia in EPO+G-CSF group were significantly better than those in the other groups (Fig. 4E, F and G). In addition, the EPO + G-CSF-treated group had a much higher strength than the other groups in terms of forelimb grip strength 28 d post-treatment relative to pre-treatment (Fig. 4H).

To verify whether subcutaneous EPO+G-CSF administration could enhance metabolic activity, we examined cortical glucose metabolism by [^18^F]fluoro-2-deoxyglucose positron emission tomography (FDG-PET) one week post-treatment (each group *n = *8). The results indicate that FDG uptake in the right cortexes of the EPO + G-CSF group was significantly better than that in the EPO, G-CSF or control groups (Fig. 4I).

Next, we examined the anti-apoptotic effects and neurotrophic factor synthesis of EPO + G-CSF in post-cerebral ischemic rats by Western blot analysis and ELISA (each group *n = *8). The anti-apoptotic protein Bcl-2 was significantly upregulated in EPO+G-CSF, EPO, and G-CSF groups, compared with control group (Fig. 4J), and the EPO+G-CSF combination induced significantly greater expression of Bcl-2 than EPO or G-CSF alone 24 hr after ischemia (Fig. 4J). One week after ischemia, rat brains treated with EPO+G-CSF had higher BDNF and SDF-1 titers than those treated with EPO alone, G-CSF alone, or control (Fig. 4K).

As measured by ELISA 3 days after G-CSF (250 µg/kg) or EPO (5000 U/kg)+G-CSF (250 µg/kg) injection, serum EPO levels in wild-type mice (C57BL/6 mice) increased significantly compared to control mice (each group *n = *6) (Fig. 4K). In contrast, G-CSF or EPO+G-CSF injection did not stimulate the additive upregulation of serum EPO in the Tg-HIF-1α^+f/+f^ mice (Fig. 4K).

Cellular apoptosis in ischemic rat brain slices was studied by TUNEL staining. Rats without MCA ligation had almost no TUNEL positive staining in their brain sections (each group *n = *8). The penumbral region surrounding the ischemic cores of EPO+G-CSF-treated rats contained significantly fewer TUNEL positive cells than the same regions in EPO alone, G-CSF alone, or ischemic control (Fig. 4L).

### EPO+G-CSF Enhanced Stem Cell Biological Activity

**Figure 5:**
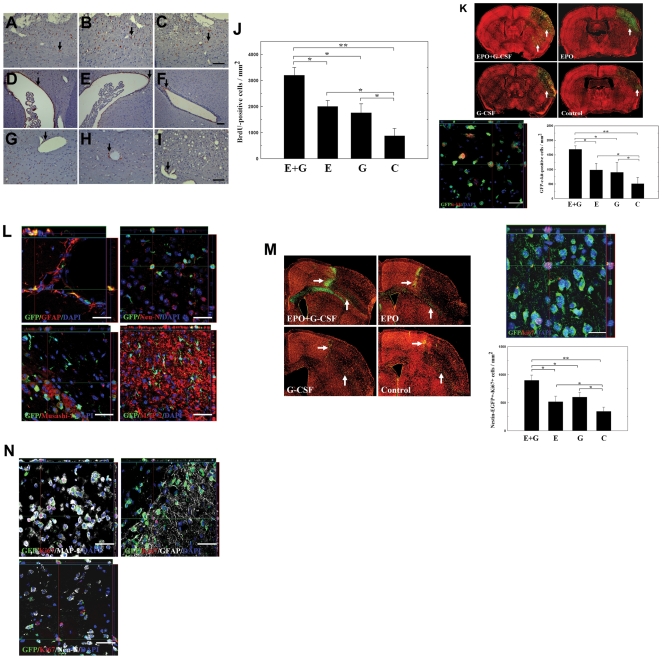
Subcutaneous administration of EPO+G-CSF enhanced the proliferation, differentiation and migration of stem cells in rats and mice. At one week after cerebral ischemia, BrdU immunoreactive cells were detected in the ipsilateral cortex near the infarct boundary (A-C, arrows), the subventricular area (D-F, arrows), and around blood vessels (G-I, arrows). (J) Numbers of BrdU immunoreactive cells were measured in the ipsilateral hemisphere of rats' brains treated with EPO+G-CSF (E+G), EPO (E), G-CSF (G) or controls (C). (K) GFP^+^c-Kit^+^ bone marrow stem cells (BMSCs) in the peri-infarct and striatal areas (white arrows) were analyzed in transgenic GFP-chimeric mice treated with EPO+G-CSF (E+G), EPO (E), G-CSF (G) or controls. (L) In double immunofluorescent analysis (3D image), many GFP^+^ cells colocalized with specific markers GFAP, Neu-N, Musashi-1 and MAP-2. (M) Nestin-EGFP^+^ INPCs (white arrows) were stained for Ki67 in the penumbral region of nestin-EGFP mice treated with EPO+G-CSF (E+G), EPO (E), G-CSF (G) or controls. (N) Nestin-EGFP^+^-Ki67^+^ cells also co-localized with specific markers MAP-2, GFAP, and Neu-N. Mean ± SEM, *P<0.05 and **P<0.01 vs. control. Bar  = 50 µm.

Bromodeoxyuridine (BrdU) labeling was used to trace the homing and engraftment of stem cells (including intrinsic neural progenitor cells [INPCs] and bone marrow derived stem cells [BMSCs]) to brain tissue at 7 days after cerebral ischemia. According to cumulative BrdU labeling immunohistochemistry, many BrdU^+^ cells were found in the ipsilateral cortex near the infarct boundary (Fig. 5A, B and C) and the subventricular region of the ischemic hemisphere (Fig. 5D, E and F). BrdU^+^ cells were also identified around the lumen of blood vessels of varying calibers in the perivascular portion of the ischemic hemisphere (Fig. 5G, H and I). Results from BrdU pulse labeling experiments showed significantly greater numbers of BrdU^+^ cells in EPO+G-CSF-treated rats than those in EPO-, G-CSF-, or saline-treated rats (each group *n = *8) (Fig. 5J).

To assess specifically whether INPCs or BMSCs proliferated and differentiated into neural cells at ischemic sites, brain slices of GFP-chimeric and nestin-EGFP mice receiving each treatment type were examined by double-staining immunohistochemistry at 28 days after cerebral ischemia. In transgenic GFP-chimeric mice, more GFP**^+^**c-Kit**^+^** BMSCs were dispersed over the right striatum, hippocampus and the penumbral area in EPO+G-CSF-treated mice compared to EPO-, G-CSF-, or saline-treated mice (each group *n = *8) (Fig. 5K). Fractions of GFP^+^c-Kit^+^ cells colocalizing with specific markers MAP-2, GFAP, Neu-N, and Musashi-1 in the EPO+G-CSF-treated mice (≈4%, ≈8%, ≈6%, and ≈5%, respectively), were significantly higher than those in the EPO-treated (≈2%, ≈5%, ≈4%, and ≈2%), G-CSF-treated (≈2%, ≈6%, ≈3%, and ≈3%) and control (≈1%, ≈0.5%, ≈1%, and ≈0.5%) groups (Fig. 5L). In nestin-EGFP mice, greater numbers of nestin-EGFP^+^ cells were also Ki67^+^ over the striatum and penumbral region in EPO+G-CSF-treated mice than in other treatment and control groups (each group *n = *8) (Fig. 5M). Quantitatively, fractions of nestin-EGFP**^+^** cells colocalizing with Ki67 and the specific markers MAP-2, GFAP, and Neu-N were ≈2%, ≈3.5%, and ≈5% in the EPO+G-CSF-treated mice, respectively, significantly higher than in the EPO-treated (≈1.3%, ≈2%, and ≈3%), G-CSF-treated (≈1%, ≈1.8%, and ≈2.5%) and control (≈0.5%, ≈0.3%, and ≈0.5%) groups (Fig. 5N).

### EPO+G-CSF Promoted Angiogenesis in Ischemic Model

**Figure 6:**
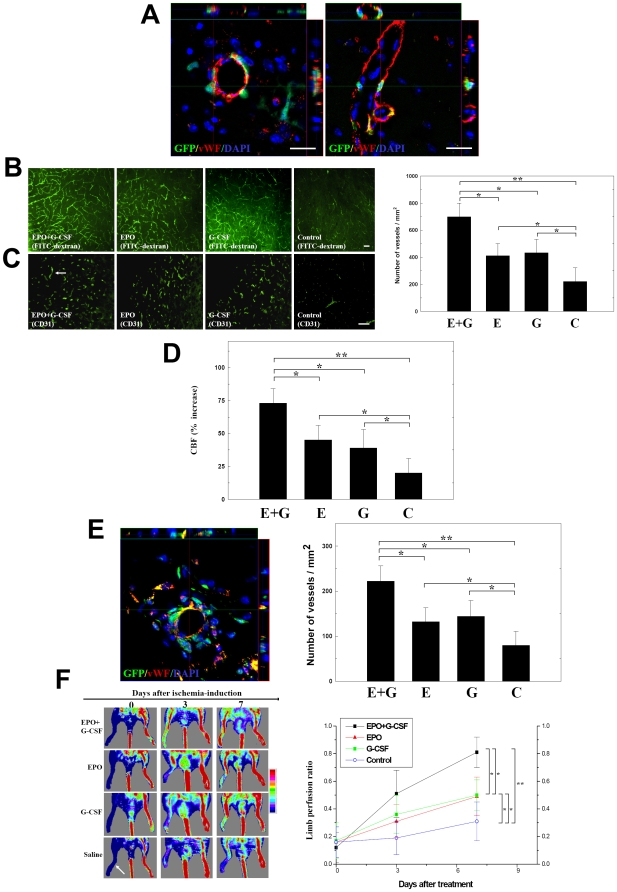
EPO+G-CSF treatment induced angiogenesis. (A) Colocalization of GFP^+^ and vWF^+^ cells in transgenic GFP-chimeric mice treated with EPO+G-CSF was analyzed in the perivascular and endothelial regions of the ischemic hemispheres. (B) Representative images of FITC-dextran perfused vessels in ischemic brain following EPO+G-CSF, EPO, G-CSF-treated and control rats. (C) Representative images and quantitative analysis of the cerebral blood vessel density after staining by CD31 immunoreactivity (white arrow) were analyzed from ischemic brain in the EPO+G-CSF, EPO, or G-CSF-treated and control rats. (D) Cerebral blood flow (CBF) was measured by laser doppler flowmetry (LDF) in the ischemic cortex treated with EPO+G-CSF, EPO, G-CSF or controls. (E) In 3D images, GFP^+^ cells colocalized with vascular phenotype vWF^+^ cells in the perivascular region of the ischemic limb muscles in hindlimb ischemic mice model. CD31-immunoreactive blood vessel density was quantified in the limb muscles treated with EPO+G-CSF, EPO, G-CSF or controls. (F) Representative images of blood perfusion in ischemic limbs were analyzed by laser doppler perfusion imaging (LDPI) analysis in mice treated with EPO+G-CSF, EPO, G-CSF or controls. Mean ± SEM, *P<0.05 and **P<0.01 vs. control. Bar  = 50 µm.

To determine whether subcutaneous EPO+G-CSF administration could induce angiogenesis through encouraging the homing of BMSCs and their differentiation into vascular-endothelial cells at ischemic sites, double-staining immunohistochemistry, FITC-dextran perfusion studies and blood vessel density assays were performed on each brain slice from each experimental mouse at 28 days after cerebral ischemia. Several GFP^+^ cells from transgenic GFP-chimeric mice showed vascular phenotypes (vWF^+^ cells) around the perivascular and endothelial regions (Fig. 6A) of the ischemic hemispheres of EPO+G-CSF-treated rats. According to a visual inspection of the FITC-dextran perfusion, much greater cerebral microvascular perfusion occurred in mice treated with EPO + G-CSF than mice in the EPO, G-CSF or control groups (each group *n = *8) (Fig. 6B). In addition, results from blood vessel density quantification by CD31 immunoreactivity (Fig. 6C) show better neovascularization in the penumbral areas of ischemic rats treated with EPO + G-CSF compared to the other three groups (each group *n = *8) (Fig. 6C).

Whether the increased blood vessel density enhanced functional CBF in the ischemic brain was examined by laser Doppler flowmetry (LDF) under anesthesia after cerebral ischemia. At one week after cerebral ischemia, CBF was significantly greater in the ischemic cortex of the EPO+G-CSF-treated rats than that in EPO, G-CSF or control (each group *n = *8) (Fig. 6D).

To determine whether subcutaneous EPO+G-CSF administration could enhance neovascularization and restore blood perfusion in peripheral limb ischemia, double staining immunohistochemistry, blood vessel density assays and laser Doppler perfusion imaging (LDPI) analysis were performed on both limbs from each experimental mouse. Several GFP^+^ cells from transgenic GFP-chimeric mice co-expressed vascular phenotypes (vWF^+^ cells) around the perivascular and endothelial regions of the ischemic limb muscles of EPO+G-CSF-treated mice (Fig. 6E). According to quantitative measurements of blood vessel density by CD31 immunoreactivity (Fig. 6E), ischemic limbs from mice treated with EPO+G-CSF had more muscle neovasculature than those with EPO or G-CSF alone or controls (each group *n = *8). In evaluation of the blood perfusion of ischemic limbs by LDPI, EPO+G-CSF treatment led to significantly greater recovery of blood perfusion of ischemic limbs than G-CSF, EPO alone or control (each group *n = *8) (Fig. 6F).

## Discussion

Although many reports have noted the synergy between EPO and G-CSF in the treatment of anemia in myelodysplastic syndromes (MDS) [16], [18], investigators did not propose any mechanism to explain the results of clinical trial. In previous reports, both EPO and G-CSF have been shown to possess anti-apoptotic, angiogenic and neuroprotective properties [2], [5], [6] and have also been regarded as therapeutic agents in acute stroke models [1], [19] and peripheral limb ischemic models [22]. In this study, we demonstrated that EPO in combination with G-CSF enhanced more plastic effects and revascularization in cerebral and limb ischemic models than either alone.

HIF-1α is activated by G-CSF in HUVECs under normoxia condition, as evidenced by our observation that G-CSF stimulation leads to enhanced HIF-1α activity and increased DNA-binding activity driven by the HRE. G-CSF is known to enhance the expression of two HIF-1α target genes, VEGF and SDF-1, in human neural stem cell cultures and platelets [22], [23]. In our study, G-CSF also enhanced expression of the HIF-1α target genes EPO in HUVECs with a similar mechanism.

Cells express many proteins in response to hypoxia and ischemia, which supports cellular adaptation in response to altered metabolic demands and the removal of toxic substances [24]. As a master regulatory gene, HIF-1α controls critical pro-angiogenic genes such as VEGF and EPO [25], [26]. Since VEGF and EPO genes contain the HRE in their promoter regions, some normoxic factors could also upregulate the expression of VEGF or EPO via HIF-1α activation [11], [27]. According to two previous studies, G-CSF may promote VEGF secretion through HIF-1α activation [28], [29]. Administration of G-CSF is known to augment angiogenesis by upregulation of VEGF in neurotrophils [28], and has been reported to raise serum VEGF level of patients [30]. Here we have demonstrated for the first time that G-CSF enhances the synthesis of EPO via upregulation of HIF-1α in primary cortical culture. The activated HIF-1α bound to the HRE of the EPO promoter and turned on the expression of EPO. The mechanism associated with EPO expression regulation and G-CSF in the present study may be similar to a previously described molecular mechanism involved in VEGF expression regulation and macrophage colony-stimulating factor (M-CSF) in cell monocytes and macrophages [29].

Recombinant EPO and G-CSF have been used clinically for over 20 years [31]. Many researchers have investigated the synergistic effects of administering EPO and G-CSF in hematopoiesis, including granulopoiesis and erythropoiesis [13], [14], [15]. Although either EPO or G-CSF has been shown to express neuroprotective and angiogenic effects in stroke animal models [1], [6], [19] and limb ischemia [22], no report has yet focused on the enhanced ability of these two cytokines in combination to induce neuroplasticity and angiogenesis. Other cytokines (stem cell factors, hepatocyte growth factors and stromal cell-derived factor 1α) in combination with G-CSF have also been reported as beneficial in animal stroke models [32], [33] and limb ischemia [34]. We showed here that G-CSF and EPO synergized to produce better functional recovery from stroke and limb ischemia than either factor alone, which might be due to much enhanced angiogenesis and significant relevant neuroprotection. Since many cytokines and growth factors may be involved in ischemic repair and recovery mechanism, additional study is required to clarify which combination therapy might have the greatest plastic effect on ischemic tissue.

In the present study, the bone marrow stem cells (BMSCs) homing and differentiation were significantly enhanced by EPO in combination with G-CSF treatment. Previous investigators have also demonstrated the individual capability of EPO or G-CSF to stimulate proliferation and differentiation of neural stem/progenitor cells inside the animal brain [6], [33], [35], [36], [37], [38], [39]. Some studies discovered that EPO enhanced the expression of a signaling protein (SOCS2) and a transcriptional factor (neurogenin 1) to regulate the neural stem/progenitor cell proliferation and differentiation [35], [37]. In other words, it is clear that both EPO and G-CSF are capable of stimulating neurogenesis to repair injured neural tissue. Other research teams have reported that a combination of G-CSF and stem cell factor (SCF) may be capable of inducing neural progenitor cell growth, thereby producing an enhanced plastic effect during brain repair [33], [39]. Here we found that a combination of G-CSF and EPO could synergistically promote proliferation of the neural progenitor cells residing in the hippocampus and subventricular region of brain. In addition to BrdU labeling methods, we further applied the Nestin-EGFP transgenic mice model to certify the proliferation, differentiation and mobilization of endogenous neural progenitor cells in the ischemic brain after a combination of G-CSF and EPO treatment. In summary, EPO in combination with G-CSF might be a feasible therapeutic strategy to treat different neurodegenerative diseases.

## Materials and Methods

### Measurement of Serum EPO Levels in G-CSF-Treated Human Donors

We obtained serum samples from age-matched (Age  = 35–40 y), healthy donors (male:female  = 12∶12, no athlete) at sequential time points (1, 3, 7 and 14 days) after subcutaneous G-CSF administration (10 to 15 µg/kg, Kirin) for 5 consecutive days according to the manufacture's instruction. All protocols and informed consents procedures were fully reviewed and approved by the Institutional Review Board of China Medical University Hospital. Informed consent was obtained from all participants. As a positive control, known level of EPO protein in serial dilution were measured using a human EPO ELISA kit (R&D Systems), with the results used to plot a standard curve. Each serum sample for EPO concentration measurement was run in triplicate and compared with the standard curve.

### Cell Culture

Human umbilical vein endothelial cells (HUVECs; Cambrex) were cultured in EGM-2 medium containing human epidermal growth factor, hydrocortisone, human fibroblast growth factor, vascular endothelial growth factor, ascorbic acid, gentamicin, amphotericin-B, human insulin-like growth factor, heparin and 2% FBS at 37°C in a 5% CO_2_ humidified air incubator as previously described [40]. Confluent cells at passage 4 were used for all experiments.

### ELISA Measurement of Activated HIF-1α in HUVECs

In order to evaluate the non-hypoxic activator effect of G-CSF, the levels of activated HIF-1α protein after G-CSF treatment were measured by ELISA. HUVECs were treated with different doses (0.01, 0.1, 1, and 10 µg/mL) of G-CSF (Kirin) for 6 hours. Nuclear extracts from these cells were incubated (50 µg/well) with biotinylated double-stranded oligonucleotide containing a consensus HIF-1α binding site from an active HIF-1α DuoSet IC ELISA kit (R&D Systems) according to the manufacturer's instructions. The level of activated HIF-1α was measured by absorbance and was expressed as optical density (OD) at 450 nm–540 nm as previously described [41]. To confirm the role of HIF-1α in the upregulation of EPO by G-CSF on HUVECs, cells were pretreated with the HIF-1α inhibitor 2-ME2 (5 µM; Sigma) for 16 h as previously described [41]. Unless otherwise mentioned, experiments were performed in triplicate.

### Western Blot and Immunocytochemical Analysis

HIF-1α and EPO expressions in HUVECs were measured by Western blot and immunohistochemical analyses. HUVECs were treated with 1 µg/mL G-CSF for different times (0.5, 1, 4, 12 and 24 hours), or treated with different concentrations of G-CSF ranging from 0.01 to 10 µg/mL for 6 hours. Western blot analyses of HIF-1α, and EPO expression from HUVECs were performed after each G-CSF treatment as previously described [42] using appropriately antibodies to HIF-1α (1∶200; Novus Biologicals), EPO (1∶200; R&D Systems) and β-actin (1∶2000; Santa Cruz Biotechnology).

For HIF-1α and EPO immunostaining, HUVECs were washed with PBS and fixed for 30 minutes at room temperature in 4% paraformaldehyde as previously described [42] using specific antibody against HIF-1α (1∶200; Novus Biologicals) and EPO (1∶200; R&D Systems) conjugated with FITC for 1 hour, and then rinsed 3 times in PBS. Finally, the slides were lightly counterstained with DAPI, washed with water, and then mounted.

To prepare cellular extracts for analysis, HUVECs were harvested and washed twice with cold phosphate-buffered saline (PBS). Nuclear and cytoplasm extracts were prepared using a commercial kit (Pierce). For oxygen glucose deprivation (OGD) treatment, cells were placed in a hypoxic chamber (Bug Box, Ruskinn Technology) for 4 hours, continuously flushed with 95% N_2_ and 5% CO_2_ at 37°C to maintain pO_2_ of <1 mmHg (OM-14 oxygen monitor; SensorMedics Corporation) [42]. To induce chemical hypoxia, cells were treated in a medium containing 60–600 mM of deferoxamine (DFO, Sigma) for 16 hours that mimics hypoxic conditions [43].

### Electrophoretic Mobility Shift Assay (EMSA)

Detailed protocols to assess HIF-1α DNA binding activity using EMSA have been described previously [44]. In this study we used a pair of oligonucleotide probes (5′-agcttGCCCTAC GTGCTGTCTCAg-3′ and 5′-aattcTGAGACAGCACGTAGGG Ca-3′) corresponding to the hypoxia-response element (HRE) in the EPO gene promoter [11]. Both oligonucleotides were non-radioisotope labeled using DIG Oligonucleotide 3′-End Labeling Kit (Roche). For supershift assays, 1 µg of anti-HIF-1α antibody (Novus Biologicals) was added to the samples 1 hour prior to the addition of labeled probes.

### Transfection and Luciferase Reporter Assay

Luciferase assays and transient transfection of pEpoEm1-luc and pEpoE-luc reporter plasmids [a kind gift from Dr. LE Huang [44]] into HUVECs were performed as described previously [45].

### In Vitro Primary Cortical Cultures (PCCs) Preparation and Oxygen Glucose Deprivation (OGD) Treatment

PCCs were prepared from the cerebral cortex of gestation day 17 embryos from Sprague-Dawley rats' embryos (Animal Center, China Medical University, Taiwan) as described previously with modification [42]. In brief, PCCs were maintained under serum-free conditions in neurobasal medium (Invitrogen), supplemented with B-27 supplement (2%; Invitrogen), glutamine (0.5 mM; Sigma), glutamate (25 mM; Sigma), penicillin (100 U/ml) and streptomycin (100 mg/ml; Invitrogen Corp.). As suggested by the manufacturer, on day 4 half of the medium was removed and replaced with fresh medium without glutamate. The cultures were maintained in a humidified incubator at 37°C with 5% CO_2_. PCCs were used for experimentation on day 7. Regarding OGD treatment, cells cultured with glucose-free Earle's balanced salt solution were placed in a hypoxic chamber for 4 hours (Bug Box, Ruskinn Technology) and continuously flushed with 95% N_2_ and 5% CO_2_ at 37°C to maintain a gas phase pO_2_ of <1 mmHg (OM-14 oxygen monitor; SensorMedics), and then reoxygenated for 24 hours.

Under OGD conditions, antiapoptotic proteins (Bcl-2, Bcl-xL) and proapoptotic proteins (Bax and Bad) from PCCs were analyzed after 12 hours of each cytokine pretreatment [G-CSF (2 µg/mL)+EPO (10 U/mL), G-CSF (2 µg/mL), EPO (20 U/mL) (Amgen) or vehicle-control] by Western blot as described previously [42]. On days 3-5 following each treatment, total amounts of BDNF, GDNF, SDF-1 and VEGF in the culture medium of PCCs were measured using a DuoSet IC ELISA kit (R&D Systems) according to the manufacturer's instructions.

### In Vivo Brain Ischemia/Reperfusion

Adult male Sprague-Dawley rats (weight 250–300 g, age 6–7^th^ week) were used for this study. The rats were anesthetized with chloral hydrate (0.4 g/kg, ip) and subjected to right middle cerebral artery (MCA) ligation and bilateral common carotid artery clamping for 90 mins as previously described [19], [42]. Post-stroke infarct volumes were identical and reproducible in each experimental rat [19], [42]. All animal researches and surgical procedures were approved to perform using sterile/aseptic techniques in accordance with China Medical University Institutional Guidelines.

### Measurement of Infarct Size Using Magnetic Resonance Image (MRI)

MRI (3.0 T, General Electric) was performed on rats in order to measure the infarct size after treatment of each group as described previously [42].

### Neurological Behavioral Measurements

Behavioral assessments were performed 3 days before and 3, 7, 14 and 28 days after cerebral ischemia. The tests measured: (a) body asymmetry (b) locomotor activity, and (c) grip strength as previously described [42].

### [^18^F]Fluoro-2-Deoxyglucose Positron Emission Tomography (FDG-PET)

Experimental rats were examined using microPET scanning of [^18^F]fluoro-2-deoxyglucose (FDG) to measure relative metabolic activity by a protocol previously described [42].

### Antiapoptotic Protein Analysis and ELISA for Growth Factors In Vivo

For Western blot analysis of apoptosis-related protein expression, experimental rats were anesthetized with chloral hydrate (0.4 g/kg, ip) at 24 hours after initiation of each treatment. Expression of apoptosis-related proteins (Bcl-2, Bcl-xL, Bax and Bad) in the right cortex and striatum region was also examined in each cytokine-treated and control rat [42]. In addition, ischemic cortical and striatal areas were evacuated on ice and immediately homogenized by a plastic homogenizer, and BDNF, GDNF, SDF-1 and VEGF protein levels were measured by ELISA as previously described with minor modification [46] according to the manufacturer's instructions (DuoSet IC ELISA kit, R&D Systems). Optical density was measured using a spectrophotometer (Molecular Device Co).

ELISA assays (Quantikine, R&D Systems) were used to measure EPO levels in blood samples 1, 3, 7, and 14 d following treatment with G-CSF or G-CSF plus EPO in wild type (C57BL/6) and TgCAGGCreERTM-HIF-1α^+f/+f^ mice (Tg-HIF-1α^+f/+f^ mice carrying a loxP-flanked allele of HIF-1α, a kind gift from Dr. Johnson [47]). The results were expressed as the net levels in the serum.

### Terminal Deoxynucleotidyl Transferase-Mediated Digoxigenin-dUTP Nick-End Labeling (TUNEL) Histochemistry

Cellular apoptosis was assayed by histochemistry using a commercial TUNEL staining kit (DeadEnd Fluorimetric TUNEL system; Promega) as previously described [48].

### Bromodeoxyuridine (BrdU) Labeling and Immunohistochemistry

BrdU (Sigma), a thymidine analog that is incorporated into the DNA of dividing cells during S-phase, was used to label cells undergoing mitotic division. Labeling protocol and BrdU immunostaining procedure have been described previously [42].

### Construction of Transgenic Mice

In order to verify the enhancement of bone marrow stem cell (BMSC) and intrinsic neural progenitor cell (INPC) mobilization and homing into the infarcted brain following one of four treatments (EPO+G-CSF, EPO, G-CSF and vehicle), transgenic GFP-chimeric mice and nestin-GFP mice were built as previously reported [49], [50]. Starting one day after cerebral ischemia, experimental mice were injected subcutaneously with EPO (5000 U/kg) + G-CSF (250 µg/kg), G-CSF (250 µg/kg), EPO (10000 U/kg) or saline vehicle for 5 consecutive days.

### Laser-Scanning Confocal Microscopy for Double Immunofluorescence Analysis

The expression of cell type-specific markers in GFP^+^ cells was examined by double immunofluorescence using laser scanning confocal microscopy as previously described [42] with specific antibodies against c-Kit (1∶200; BD Pharmingen), GFAP (1∶400; Sigma), MAP-2 (1∶200; BM), nestin (1∶400; Sigma), Neu-N (1∶200; Chemicon), vWF (1∶400; Sigma), Musashi-1 (1∶300; Chemicon) and Ki67 (1∶400; Novocastra Laboratories) conjugated with FITC (1∶500; Jackson Immunoresearch) or Cy3 (1∶500, Jackson Immunoresearch). The tissue sections were analyzed with a LSM510 laser-scanning confocal microscope (Carl Zeiss).

### Evaluation of Cerebral Angiogenesis

Cerebral microcirculation was analyzed by intravenous administration of the fluorescent plasma marker FITC-dextran (Sigma), and CD31 immunohistochemical analysis as previously described [42].

### Measurement of Cerebral Blood Flow (CBF)

Rats anesthetized with chloral hydrate were positioned in a stereotaxic frame. Baseline local cortical blood flow (bCBF) was monitored with a laser doppler flowmeter (LDF monitor, Moore Instruments) as previously described [51]. In brief, CBF values were calculated as a percentage increase of bCBF.

### Mouse Hindlimb Ischemia and Laser Doppler Imaging Analysis

Transgenic GFP-chimeric mice were anesthetized with chloral hydrate (0.4 g/kg, ip). The femoral artery ligation, limb blood flow measured by laser Doppler perfusion imaging (LDPI, Moore Instruments) and immunohistochemical studies were performed as previously described [52]. One day after arterial ligation, mice were randomly assigned to one of the four treatment groups: EPO (5000 U/kg)+G-CSF (250 µg/kg), G-CSF (250 µ/kg), EPO (10000 U/kg), and saline control.

### Statistical Analysis

Observers were blind to the experimental conditions of each measurement. Results are expressed as mean ± SEM. The behavioral scores were evaluated for normality. We used one-way or two-way ANOVA with appropriate post hoc Newman-Keuls testing to evaluate mean differences between different groups with different treatments. A value of *P*<0.05 was considered as significant.
