# Correction: Granulocyte Colony-Stimulating Factor Activating HIF-1α Acts Synergistically with Erythropoietin to Promote Tissue Plasticity

**DOI:** 10.1371/annotation/433064f4-e30a-4000-8e5a-9e8d1775d820

**Published:** 2010-10-08

**Authors:** Shih-Ping Liu, Shin-Da Lee, Hsu-Tung Lee, Demeral David Liu, Hsiao-Jung Wang, Ren-Shyan Liu, Shinn-Zong Lin, Ching-Yuan Su, Hung Li, Woei-Cherng Shyu

Ching-Yuan Su and Dr. Hung Li were not included in the author byline. They should be listed as the 8th and 9th authors, respectively, and affiliated with the Institute of Molecular Biology, Academia Sinica, Taipei, Taiwan. Ms. Ching-Yuan Su's contributions are as follows: performed the experiments, analyzed the data, contributed reagents/materials/analysis tools. Dr. Hung Li's contributions are as follows: conceived and designed the experiments, analyzed the data, contributed reagents/materials/analysis tools, wrote the paper. 

